# An Integrated Linkage, Chromosome, and Genome Map for the Yellow Fever Mosquito *Aedes aegypti*

**DOI:** 10.1371/journal.pntd.0002052

**Published:** 2013-02-14

**Authors:** Vladimir A. Timoshevskiy, David W. Severson, Becky S. deBruyn, William C. Black, Igor V. Sharakhov, Maria V. Sharakhova

**Affiliations:** 1 Department of Entomology, Fralin Life Science Institute, Virginia Tech, Blacksburg, Virginia, United States of America; 2 Department of Biological Sciences, Eck Institute for Global Health, University of Notre Dame, Notre Dame, Indiana, United States of America; 3 Department of Microbiology, Immunology and Pathology, Colorado State University, Fort Collins, Colorado, United States of America; National Institute of Allergy and Infectious Diseases, United States of America

## Abstract

**Background:**

*Aedes aegypti*, the yellow fever mosquito, is an efficient vector of arboviruses and a convenient model system for laboratory research. Extensive linkage mapping of morphological and molecular markers localized a number of quantitative trait loci (QTLs) related to the mosquito's ability to transmit various pathogens. However, linking the QTLs to *Ae. aegypti* chromosomes and genomic sequences has been challenging because of the poor quality of polytene chromosomes and the highly fragmented genome assembly for this species.

**Methodology/Principal Findings:**

Based on the approach developed in our previous study, we constructed idiograms for mitotic chromosomes of *Ae. aegypti* based on their banding patterns at early metaphase. These idiograms represent the first cytogenetic map developed for mitotic chromosomes of *Ae. aegypti*. One hundred bacterial artificial chromosome clones carrying major genetic markers were hybridized to the chromosomes using fluorescent *in situ* hybridization. As a result, QTLs related to the transmission of the filarioid nematode *Brugia malayi*, the avian malaria parasite *Plasmodium gallinaceum*, and the dengue virus, as well as sex determination locus and 183 Mbp of genomic sequences were anchored to the exact positions on *Ae. aegypti* chromosomes. A linear regression analysis demonstrated a good correlation between positions of the markers on the physical and linkage maps. As a result of the recombination rate variation along the chromosomes, 12 QTLs on the linkage map were combined into five major clusters of QTLs on the chromosome map.

**Conclusion:**

This study developed an integrated linkage, chromosome, and genome map—iMap—for the yellow fever mosquito. Our discovery of the localization of multiple QTLs in a few major chromosome clusters suggests a possibility that the transmission of various pathogens is controlled by the same genomic loci. Thus, the iMap will facilitate the identification of genomic determinants of traits responsible for susceptibility or refractoriness of the mosquito to diverse pathogens.

## Author Summary

About half of the human population is under risk of dengue infection. Because of the absence of a vaccine or drug treatment, the prevention of this disease largely relies on controlling its major vector mosquito *Aedes aegypti*. Availability of the complete genome sequence for this mosquito offers the potential to help in the identification of novel disease control strategies. An efficient vector of arboviruses, *Ae. aegypti* is also a convenient model for laboratory studies. A number of genetic loci related to the remarkable ability of this mosquito to transmit various pathogens were genetically mapped to the three linkage groups corresponding to the three individual chromosomes of the mosquito. However, the exact physical positions of the genetic loci and genomic sequences on the chromosomes were unknown. In this study, we developed maps for mitotic chromosomes of *Ae. aegypti* and localized 100 clones carrying major genetic markers, which were previously used for mapping genetic loci associated with the pathogens' transmission. Finally, linkage, chromosome, and genome maps of *Ae. aegypti* were integrated. Anchoring of the genomic sequences associated with genetic markers to the chromosomes of *Ae. aegypti* will help to identify candidate genes that might be utilized for developing advanced genome-based strategies for vector control.

## Introduction

Mosquitoes are vectors of numerous human pathogens such as malaria parasites transmitted by the subfamily Anophelinae; lymphatic filarial worms transmitted by both Anophelinae and Culicinae subfamilies; and arboviruses whose transmission is largely associated with the subfamily Culicinae. *Aedes aegypti* is recognized as a principal vector of dengue and yellow fever viruses. These two diseases have a significant world-wide impact on human health. Dengue fever is currently considered the most important vector-borne arboviral disease of the 21^st^ century [1]. The disease is a threat to 3.6 billion people with an annual incidence of 230 million cases of infection resulting in 21,000 deaths per year. Since the 1950s, the incidence of dengue fever has expanded globally. The World Health Organization (WHO) estimated a 30-fold increase in the incidence of dengue infections over the past 50 years [2]. The disease became endemic in 100 countries in Africa, West Asia, and America [3] and is a growing threat to the United States [4]. In addition to dengue, yellow fever, a devastating disease of the 19^th^ century in North America and Europe, still affects up to 600 million lives and remains responsible for about 30,000 deaths annually [5]. The disease is currently endemic in 32 countries in Africa and 13 in South America. The spread of the pathogens is associated with the extremely tight connection of their major vector *Ae. aegypti* to humans. Despite all control campaigns, *Ae. aegypti* currently occupies most subtropical and tropical regions in the world.

*Ae. aegypti* represents both an efficient vector of arboviruses and a convenient model system for experimental laboratory research. This species can be easily colonized and is highly tolerant to inbreeding [6]. Unlike *Anopheles* eggs, *Ae. aegypti* eggs are resistant to desiccation and can be stored in a dry place for several months. As a result of these advantages, genetic (linkage) mapping conducted on *Ae. aegypti* was very successful. The genetic mapping was originally inspired from the study of the inheritance of **d**ichloro**d**iphenyl**t**richloroethane (DDT) resistance as a single dominant trait [7]. A similar mechanism of inheritance, as a single gene or a single block of chromosome material, was demonstrated for sex determination [8]. The sex determination alleles were described as *Mm* in males and *mm* in females and linked to homomorphic chromosome 1 [9]. In addition, 28 of 87 morphological mutations described for *Ae. aegypti* were mapped to the three linkage groups corresponding to the three chromosomes of this mosquito [10]. The linkage map was extended by additional mapping of physiological and enzyme loci [11]. The classical linkage map included about 70 loci of morphological mutants, insecticide resistance, and isozyme markers [12].

A possibility of using DNA molecular markers opened a new era in genetic mapping of mosquito genomes. The first molecular-marker-based linkage map for *Ae. aegypti* was constructed using restriction-fragment-length polymorphism (RFLP) of complementary DNA (cDNA) clones [13]. This map included 50 DNA markers and covered 134 centimorgan (cM) across the three linkage groups. Thereafter, the polymerase chain reaction (PCR) was used to generate a map based on random-amplified polymorphic DNA (RAPD) loci which consisted of 96 RAPD loci covering 168 cM [14]. Linkage maps based on single-strand conformation polymorphism (SSCP) and single nucleotide polymorphism (SNP) markers were also constructed. A composite map for RFLP, SSCP, and SNP markers incorporated 146 loci and covered 205 cM [6]. Later, an additional map using amplified fragment-length polymorphism (AFLP) was also developed for 148 loci and covered about 180 cM of the genome [15]. Finally, the genetic map of *Ae. aegypti* was extended by incorporating microsatellite loci [16]. The linkage map was used as a tool to localize several quantitative trait loci (QTLs) related to pathogen transmission: the filarioid nematode *Brugia malayi* [17], the avian malaria parasite *Plasmodium gallinaceum* [15], [18], and dengue virus [19], [20]. Among all mosquitoes, the map developed for *Ae. aegypti* is the most densely populated.

Cytogenetic mapping on *Ae. aegypti* and other culicine species is difficult due to the absence of high-quality, easily spreadable polytene chromosomes [21], [22] The majority of cytogenetic studies for *Ae. aegypti* have been conducted on mitotic chromosomes from brain ganglia or meiotic chromosomes from male testis [23]–[26]. These studies led to the conclusion that *Ae. aegypti* has a karyotype consisting of three pairs of metacentric chromosomes [27]. The chromosomes first numbered as chromosomes I, II, and III in order of increasing size [23] were later renumbered as chromosomes 1, 2, and 3 in correspondence to the linkage map developed for *Ae. aegypti*, resulting in the longest chromosome III becoming chromosome 2 [9]. Chromosomes from brain ganglia of *Ae. aegypti* were first utilized for the successful nonfluorescent *in situ* hybridization of two ribosomal genes [28]. Fluorescent *in situ* hybridization (FISH) technique has been developed using mitotic chromosomes from the ATC-10 cell line of *Ae. aegypti*, resulting in direct positioning of 37 cosmid clones onto chromosomes [29]. In addition, 21 cDNA genetic markers and 8 cosmid clones containing the RFLP markers have been mapped to the chromosomes from this line [30]. This map was the first attempt to integrate linkage and physical maps for *Ae. aegypti*.

The genome of *Ae. aegypti* was among the first three mosquito genomes sequenced in the last decade [31]. As compared with *Anopheles gambiae* [32] and *Culex quinquefasciatus* [33] genomes, the genome of *Ae. aegypti* is the largest and consists of 1,376 Mb. The availability of the *Ae. aegypti* genome provides an opportunity to integrate linkage, chromosome, and genome maps for this mosquito. A total of 106 bacterial artificial chromosome (BAC) clones carrying major genetic markers have been identified by screening an *Ae. aegypti* BAC library prepared from the Liverpool strain [34]. In addition, a new cytogenetic approach based on mitotic chromosomes from imaginal discs (IDs) of 4^th^ instar larvae has been recently developed [35]. Instead of using cell lines, which usually accumulate chromosomal rearrangements [36], this method utilizes live larvae for cytogenetic analysis. A preparation slide of one ID contains ∼175 chromosome spreads. This number is 6-fold greater than that of two brain ganglia. Clearly visible banding patterns of mitotic chromosomes from IDs allowed the construction of preliminary idiograms without numbered divisions for the chromosomes at mid-metaphase [35]. A FISH technique was optimized for using BAC clones as probes [37] resulting in the assignment of 10 BAC clones and ribosomal 18S DNA to bands on the idiograms [35].

In the current study, we constructed new idiograms for the longer early-metaphase chromosomes with numbered divisions and subdivisions. These idiograms facilitated assignment of 100 BAC clones carrying major genetic markers to chromosomal bands. BAC clones within each band on the chromosomes were additionally ordered based on multicolor FISH on higher resolution prophase or polytene chromosomes. Finally, because each BAC clone also represents a supercontig in the *Ae. aegypti* genome assembly, the total of 183 Mb or 13.3% of the genomic sequences was also incorporated into the map. We define our map as integrated linkage, chromosome, and genome map or an iMap of *Ae. aegypti*.

## Methods

### Mosquito strain

This study was performed on the Liverpool IB12 strain of *Ae. aegypti*, which was previously used for the genome sequencing project [31]. This strain originated from the Liverpool strain (LVP) following several rounds of inbreeding. LVP was a major strain for conducting genetic and QTL mapping in the past [13], [17], [18]. Originally, mosquitoes for this strain were collected in West Africa and then kept by the Liverpool School of Medicine [38].

### Chromosome preparation

Chromosome preparations were prepared from imaginal discs (IDs) or salivary glands of the 4^th^ instar larvae of *Ae. aegypti* (Timoshevskiy et al., 2012). For ID dissection larvae were placed on ice for several minutes for immobilization. Individual larva was decapitated in a drop of cold hypotonic solution (0.5% sodium citrate). Then the thoracic part of the larva was dissected, and cuticle from the ventral side of the larval thorax was cut by dissecting scissors (Fine Science Tools, USA) and opened. The gut and fat body particles were removed, and a new drop of hypotonic solution was applied. After 10 min., the hypotonic solution was removed using filter paper, and a drop of fixative solution (ethanol/acetic acid in 3∶1 ratio) was applied. IDs were isolated using dissecting needles (Fine Science Tools, USA), placed in a drop of 50% propionic acid for maceration, and squashed under a cover slip (22×22 mm). Salivary glands were dissected from the larvae prefixed in fixative solution (ethanol/acetic acid in 3∶1 ratio) for at least 24 hrs and then squashed in a drop of 50% propionic acid. Presence of chromosomes on the slide was determined by using a phase-contrast microscope Olympus CX41 (Olympus America, Inc., USA) at 200× magnification. Slides suitable for further applications were placed in liquid nitrogen and cover slips were removed. Finally, slides were dehydrated in an ethanol series (70, 80, 100%) and stored at −20°C.

### Probe/C_o_t fraction DNA preparation

BAC clone DNA was prepared by the Clemson University Genomics Institute in 96-well plates. For the probe preparation, BAC clone DNA was labeled by nick-translation. The reaction mix with final volume of 25 µl contained 0.5 µg of DNA, 0.05 mM each of unlabeled dATP, dCTP, and dGTP, and 0.015 of mM dTTP, 0.5 µl of Cy3-, or Cy5-dUTP (GE Healthcare UK Ltd, Buckingham-shire, UK), or 1 µl fluorescein-12-dUTP (Fermentas, Inc., USA), 0.05 mg/ml BSA, 2.5 µl of 10× nick translation buffer, 10 u of DNA polymerase I, and 0.0006 u of DNAse I (Fermentas, Inc., USA). DNA polymerase/DNAse ratio was selected empirically to obtain the probe with a size range from 300 to 500 base pair. For performing FISH with additional colors (besides Cy3, Cy5, and fluorescein), a pair combination of equal volumes of differently labeled probes was used.

Optimized methods for isolation of the repetitive DNA fraction for *Ae. aegypti* was described by Timoshevskiy et al., 2012. Genomic DNA was isolated from adult mosquitoes using Qiagen Blood & Cell Culture DNA Maxi Kit (Qiagen Science, USA). For individual extractions, approximately 500 mg of adult mosquitoes were taken. For further manipulation, DNA was dissolved in 1.2× SSC buffer to final concentration of 1 µg/µl. For shearing, a denatured DNA solution was heated at 120°C for 2 min. Reassociation of the DNA was performed at 60°C for 10 min or 15 min. After reannealing, samples were placed in ice, and 10× S1-nuclease buffers and S1 nuclease (100 U per 1 mg DNA) were added. Nuclease treatment was performed at 42°C for 1 hr. Isolated repetitive DNA fractions were precipitated by isopropanol and dissolved in TE-buffer. According to our estimation, the repetitive fractions isolated using this approach correspond to C_o_t2 or C_o_t3 DNA fraction and contained all highly repetitive and part of medium-repetitive DNA fragments (Trifonov et al., 2009). Final outcome of C_o_t DNA fraction accounts for ∼30% of the *Aedes* genomic DNA. Repetitive DNA fractions of genomic DNA were utilized to suppress repetitive sequences in hybridizations to the chromosomes.

### FISH

*In situ* hybridization was performed using a modified standard human protocol (Timoshevskiy et al., 2012). Slides were placed in 2× SSC for 30 min at 37°C, pretreated with 0.1 mg/ml of pepsin for 5 min at 37°C, denaturated in 70% formamide in 2× SSC at 72°C for 2 min, and dehydrated in a series of cold (−20°C) ethanol (70, 80, 100%) for 3–5 min each. Hybridization mix contained: 50% formamide, 10% dextran sulfate, 100 ng of each probe per slide, and 3 µg of unlabeled repetitive DNA fractions per probe. DNA/probe mix was precipitated by adding 1/10 volume of sodium acetate and 2 volumes of 100% ethanol. The DNA pellet was dissolved in “master mix” (10 µl per slide) that contained 50% formamide, 10% dextransulfate, and 1.2× SSC. After that, DNA was denatured at 96°C for 7 min. Denatured DNA was placed on ice for 1 min and incubated at 37°C for 30 min for pre-hybridization with unlabeled repetitive DNA fractions. Ten µl of hybridization mix was placed on a slide, which had been preheated to 37°C, under a 22×22 mm cover slip, and glued by rubber cement. Slides were hybridized at 37°C in a dark humid chamber overnight. After hybridization, slides were dipped for washing in a Coplin jar with 0.4× SSC, 0.3% Nanodept-40 at 72°C for 2 min, and then in 2× SSC, 0.1% Nanodept-40 at RT for 5 min. Thereafter, slides were counterstained using Prolong with DAPI (Invotrogen Corporation, USA) or incubated with 1 µM YOYO-1 solution in 1× PBS for 10 min in the dark, rinsed in 1× PBS, and then enclosed in antifade Prolong Gold (Invitrogen Corporation, USA) under a cover slip. Slides were analyzed using a Zeiss LSM 510 Laser Scanning Microscope (Carl Zeiss Microimaging, Inc., USA) at 1000× magnification.

### Image processing

To develop idiograms, the best images of the chromosomes from IDs stained with YOYO-1 were selected. The colored images were converted into black and white images and contrasted in Adobe Photoshop as described previously [39]. The chromosomal images were straightened using ImageJ program [40] and were aligned for comparison. In total, ∼90 chromosomes at early metaphase were analyzed. FISH images were also filtered using ImageJ program [40]. For ordering genetic markers, chromosomes at various levels of condensation were used. Prometaphase and early metaphase chromosomes were utilized for assigning genetic markers to the particular chromosome bands. Prophase and polytene chromosomes were used for ordering markers within the same chromosome band. From 10 to 20 images were analyzed to obtain reproducible ordering patterns.

### Measurements and statistics

Chromosomes were measured as described previously [34] using Zen 2009 Light Edition software [41]. The relationship between the physical locations of markers and their linkage positions was assessed by assigning genes of known physical position an integer score. These scores were 1–26 (1p3.4–1q4.4) on chromosome 1, 1–36 (2p4.4–2q4.4) on chromosome 2, and 1–32 (3p4.4–3q4.4) on chromosome 3. This integer score was then regressed upon the cM position of the gene as determined in a number of previous independent linkage mapping studies and F_1_ intercross families. Linear regression analysis was performed using R (2.14.1) [42]. These regression models were then used to predict the physical position of the markers for which we have linkage positions in cM.

## Results and Discussion

### Cytogenetic map of mitotic chromosomes of *Ae. aegypti*

**Figure 1:**
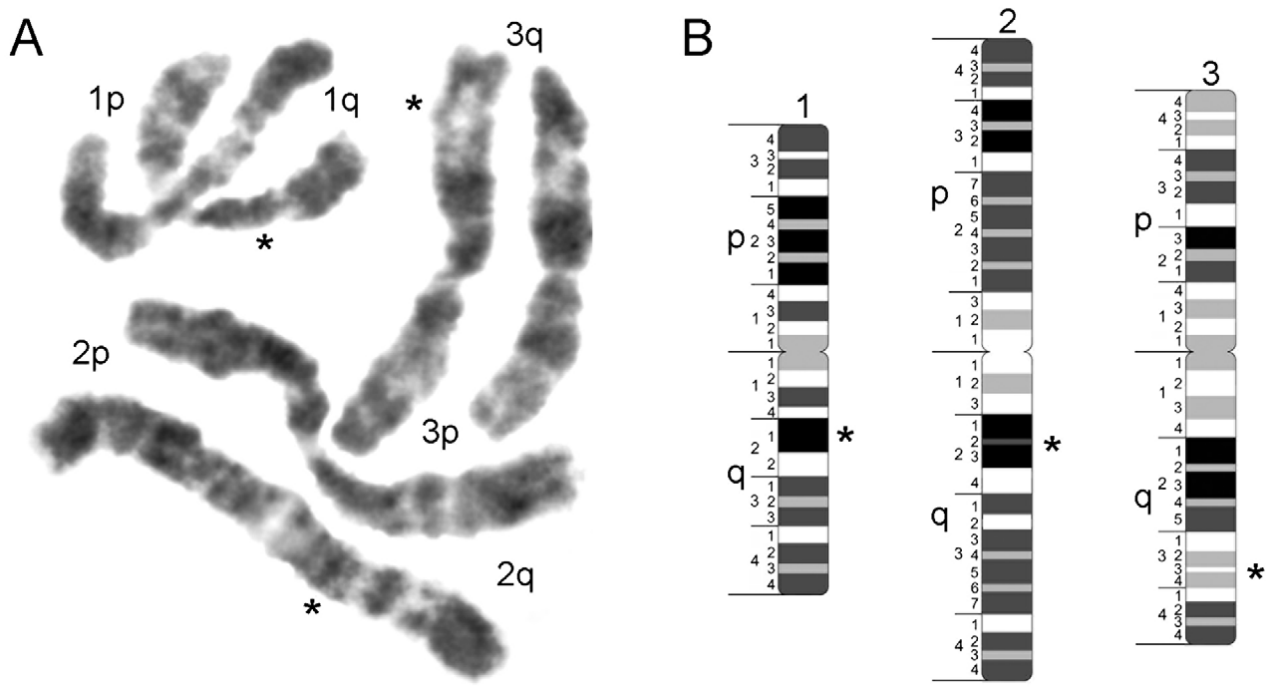
The construction of idiograms for mitotic chromosome of *Aedes aegypti*. Early metaphase chromosomes of *Ae. aegypti* stained with YOYO-1 iodide (A) were utilized for idiogram (B) development. Chromosome numbers are shown on the top of each chromosome. Chromosomal arms p and q and numbered divisions and subdivisions are shown on the left side of the idiograms. Landmarks are indicated by asterisks.

Our previous study developed preliminary idiograms – diagrammatic representations of the chromosome banding patterns – for the mid-metaphase chromosomes of *Ae. aegypti* [35]. This stage of mitosis is the most representative in any chromosome preparation. At mid metaphase, chromosomes and chromosome arms can be easily distinguished from each other based on their length and presence of specific landmarks. Chromosomes on preliminary idiograms were not divided into divisions and subdivisions, and these idiograms served only for chromosome and chromosome arm identification. In the current study, we developed idiograms for the chromosomes of *Ae. aegypti* at an earlier stage of mitosis – early metaphase. The average chromosome lengths at this stage are 11.86, 16.19, and 13.64 µm for chromosomes 1, 2, and 3, respectively, or ∼5.5 µm longer than at mid metaphase. At stages of mitosis previous to metaphase, such as prophase and prometaphase, homologous chromosomes of *Ae. aegypty* are usually tightly paired [35]. Although chromosomes at these stages are longer than at early metaphase, the banding patterns of the chromosomes are variable. At early metaphase, chromosomes finally segregate from each other, resulting in a visible number of chromosomes becoming equal to 6 and chromosome patterns becoming more reproducible (Figure 1A). We consider this stage of mitosis as the most reliable for the development of the chromosome map, which can be used for the detailed physical mapping.

Similarly to our previous study [35], we used chromosome images stained with YOYO-1 iodide. This dye provides much clearer banding patterns as compared with the most commonly used DAPI (4′,6-diamidino-2-phenylindole fluorescent stain). Images of the chromosomes were converted into black and white images (Figure 1A) and straightened. Similarly to the idiograms of human chromosome [43], we identified chromosome bands of 4 different intensities – intense, medium intensity, low intensity, and negative (Figure 1B). Chromosomes were finally subdivided into 23 numbered divisions and 94 bands. The following regions can be considered as landmarks for the chromosome arm recognition: intense band in division 1q21; intense double band in divisions 2q21–23; and 2 low-intensity bands in region 3q32, 3q34. These regions are shown by asterisks on Figure 1. Large negative bands indicate the boundaries between all divisions on the chromosomes.

Chromosome idiograms constructed in this study represent the first cytogenetic map developed for mitotic chromosomes of *Ae. aegypti*. In studies conducted before on chromosomes from cell lines of *Ae. aegypti*, the positions of the markers on the chromosomes were measured by FLpter: a fractional length from the short-arm telomeric end p-terminus [30]. As a result, this mapping provides only approximate coordinates for the markers. The idiograms recently developed for mid-metaphase chromosome [35] were designed mostly for individual chromosome and chromosome arm recognition. The map for early metaphase chromosome presented here is designed for the more detailed band-based mapping. It finally permits assignment of the location of the specific DNA signals to the particular numbered subdivision on the chromosomes.

### A “two-step” approach to physical mapping

**Figure 2:**
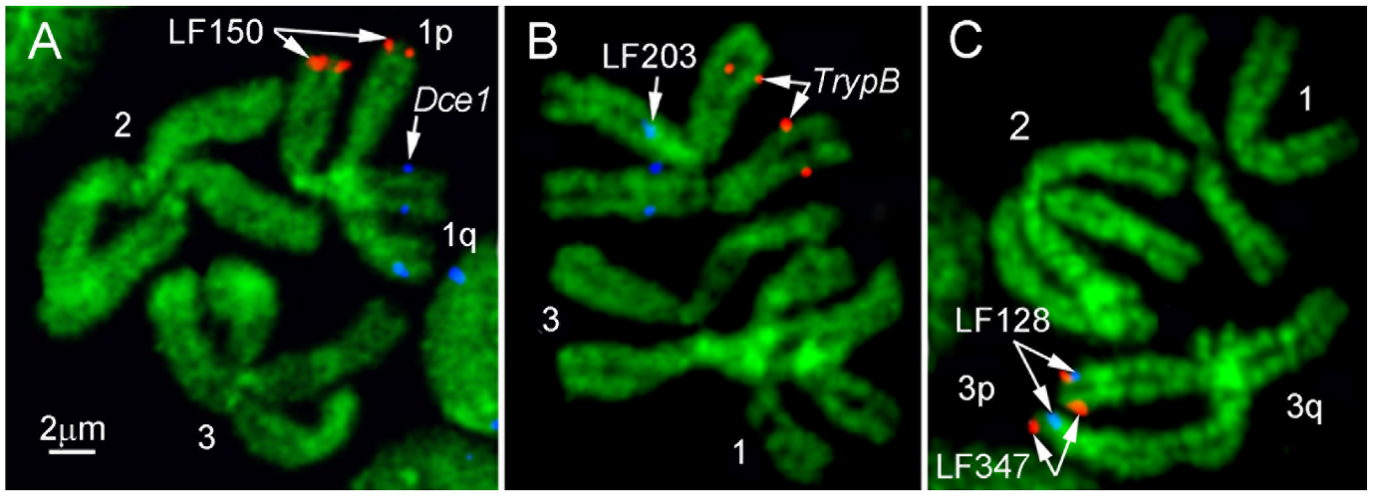
FISH on early metaphase chromosomes of *Aedes aegypti*. BAC DNA probes labeled by CY3 (red) and Cy5 (blue) were hybridized to the chromosomes stained with YOYO-1 iodide. Positions of the BAC clones on chromosomes 1 (A), 2 (B), and 3 (C) are indicated by arrows.

Previous efforts identified 106 BAC clones that carry genetically mapped marker sequences [34]. We used a “two-step” physical mapping approach for 1) assigning BAC clones to the chromosomal bands and 2) ordering them within the band. For the first step, we utilized FISH on chromosomes stained with the green dye YOYO-1 iodide. BAC clone DNA was labeled with Cy3 (red) and Cy5 (infrared) fluorescent dyes. Examples of FISH results on early metaphase chromosomes are shown in Figure 2. Each FISH allowed us to place two BAC clones to a specific band on idiograms. Eight BAC clones produced more than one hybridization signal. In these cases, the most intense signal was considered as a major position of the BAC clone on the chromosomes. In total, 100 out of 106 BAC clones were successfully assigned to specific bands on *Ae. aegypti* chromosomes (Table 1).

**Table 1:** Chromosome locations of genetic markers and genomic supercontigs.

Clone	LG #	Accession #	Supercontig (SC)	BAC Plate/Well	SC size (bp)	Chromosome location
AEGbS11	1	AY033622	SC1.68	NDL 017, O21	2950385	1q44
AEGI22	1	BI099650	SC1.438	NDL 054, F-19	1121788	1q44
*AeW*	1	U73826	SC1.71	NDL 058, C-3	2873990	1p14
*AmyII*	1	AF000568	SC1.326	NDL 058, D-8	1381451	1q12
*CHT2*	1	AF026492	SC1.673	NDL 129, I-16	598027	1q13
D6L500	1	BH214541	SC1.153	NDL 059, H-24	1723990	1q33
*Dce1*	1	AF288384	SC1.1	NDL 008, F-24	5856339	1q33*, 2 signals on chr1
*FerH*	1	AF326341	SC1.252	NDL 035, O-21	1852562	1q41
*LAP*	1	M95187	SC1.192	NDL 018, P-1	1864021	1p13
LF150	1	BM005476	SC1.777,1.12,1.52,1.684	NDL 088, D-2	2908877	1p33
LF159	1	T58315	SC1.388	NDL 073, M-10	1135229	1q21
LF178	1	T58309	SC1.59	NDL 033, C-3	3045158	1p31*, 2p44
LF179	1	BM005479	SC1.123	NDL 017, H-6	2410060	1p12
LF198	1	T58319	SC1.710	NDL 090, O-23	643802	1p32
LF204	1	BM378050	SC1.415,1.142	NDL 044, O-22	1112054	1p21
LF217	1	BM005473	SC1.50	NDL 009, N-2	768001	1p33
LF284	1	BM005502	SC1.446	NDL 062, O-7	1002973	1q21
LF314	1	BM005509	SC1.123	NDL 100, G-8	2410060	1p12
LF90	1	T58320	SC1.148	NDL 088, A-20	2163576	1p34
*nAcBP*	1	AY040341	SC1.1051,1.465	NDL 109, E-9	322300	1q42
*NaK*	1	AF393727	SC1.650	NDL 050, A-4	647669	1q42
rDNA 18S	1	AY988440	SC1.836	NA	436213	1q22
RT6	1	BH214544	SC1.440,1.1595	NDL 060, C-14	967717	1q44
*SiaI*	1	AF108099	SC1.4	NDL 085, K-2	5177111	1p34
*slo*	1	AF443282	SC1.96	NDL 010, B-1	2767225	1q33
*Tsf*	1	AF019117	SC1.2392,1.4036,1.176	NDL 013, I-5	111500	1p33
TY7	1	R19560	SC1.88	NDL 046, L-1	2707514	1q32
A13L975	2	BH214533	SC1.5	NDL 044, N-13	5058281	2p34
a14	2	BH214531	SC1.259	NDL 064, G-23	1579804	2p11
AEGI10	3	BI096854	SC1.290	NDL 045, K-4	292900	2q22
*AEG12*	2	AY038041	SC1.477,1.875	NDL 067, K-5	943092	2q31
AEGI23	2	AY033624	SC1.377	NDL 061, B-12	211600	2p32
AEGI27 (LF181)	2	BG937399	SC1.151	NDL 118, J-5	2103471	2p41
AEGI8	2	AF326340	SC1.145	NDL 106, A-1	2217386	2p44
*AmyI*	2	AF000569	SC1.219	NDL 020, N-18	1777957	2p34
ARC1	2	R19561	SC1.14	NDL 060, L-12	4341222	2q42*, multiple signals
B8M980	2	BH214534	SC1.316	NDL 036, I-21	1322148	2q37
BA67 (Sin3)	2	AI561370	SC1.1132,1.1232	NDL 046, O-19	217800	2q44
*Chym* (LF173)	2	AY038039	SC1.76,1.1028	NDL 052, J-18	2906033	2q33
*CRALBP*	2	AF329893	SC1.29	NDL 088, D-9	3855786	2q42
D6L600	2	BH214535	SC1.14	NDL 022,N-19	4341222	2q42
*D7*	2	M33156	SC1.204	NDL 048, J-19	1827752	2q36
F17M590	2	BH214537	SC1.213	NDL 025, H-18	1750228	2q44
*GS1*	2	AF004351	SC1.45	NDL 018, I-7	3214345	2q35
LF115	2	R67978	SC1.328	NDL 044, H-3	1305728	2p42
LF129	2	BM005504	SC1.816	NDL 079, D-19	517624	2q33
LF138	2	T58332	SC1.25	NDL 055, C-23	3904351	2p21
LF158	2	BM005485	SC1.1168	NDL 040, I-24	191961	2p11
LF169	2	BM378049	SC1.701	NDL 091, P-10	988662	2p12
LF180	2	BM005486	SC1.44	NDL 096, I-16	3232429	2p13
LF203	2	BM005503	SC1.157	NDL 045, M-21	2064759	2q21
LF211	2	BM005514	SC1.209	NDL 045, E-5	1779872	2q44
LF223	2	BM005515	SC1.275	NDL 098, E-9	1521713	2q44
LF233	2	T58327	SC1.132	NDL 008, I-12	2355619	2p32
LF250	2	T58310	SC1.581	NDL 041, P-5	760982	2p42
LF275	2	BM005500	SC1.507	NDL 036, A-1	845243	2q23
LF282	2	T58328	SC1.277	NDL 097, L-13	1632801	2p32
LF291	2	BM005482	SC1.786,1.163	NDL 038, M-5	488859	2p42
LF335	2	BM005505	SC1.244	NDL 044, A-23	1610334	2q41
LF342	2	BM005512	SC1.39,1.348	NDL 026, O-21	3590018	2p33
LF357	2	BM005495	SC1.48	NDL 111, G-22	3355344	2q33
LF407	2	BM005510	SC1.219	NDL 015, J-1	1777957	2p34
LF409	2	BM005511	SC1.704	NDL 002, K-24	585311	2p34
LF98	2	T58313	SC1.148	NDL 030, J-23	2163576	2p41
*MUCI* (LF398)	2	AF308862	SC1.58,1.130	NDL 008, H-13	3090966	2p32
*Rdl*	2	U28803	SC1.319	NDL 014, P-21	1366586	2p43
*RpL17A* (LF355)	2	AF315597	SC1.875,1.1393,1.789	NDL 013, I-3	466421	2q31*, multiple signals
*SDR* (BS2)	2	AY033621	SC1.456	NDL 039, N-24	945809	2q13
*Sec61*	2	AF326338	SC1.122,1.500	NDL 052, E-23	2401221	2q24
*TrypB*	2	M77814	SC1.817	NDL 033, H-12	451076	2p33
*VCP*	2	L46594	SC1.210+8 more	NDL 061, G-1	2048554	2p42
*VMP15a-3*	2	U91682	SC1.216,1.1166	NDL 041, C-6	1816664	2q31
a12	3	BH214530	SC1.206	NDL 057, G-20	1835550	3q13
*apoLp-II*	3	AF038654	SC1.441	NDL 073, A-24	1009977	3p32
*Apy1*	3	L12389	SC1.201	NDL 116, G-24	1818773	3q44
*CYP9J2*	3	AF329892	SC1.221	NDL 089, M-4	1752526	3q41
*def*	3	AF156088	SC1.98	NDL 051, L-10	2816416	3q32*, multiple signals
LF103	3	BM005488	SC1.766	NDL 005, F-19	506593	3q11
LF106	3	BM005490	SC1.1, 1.488	NDL 067, B-23	5856339	3p23
LF108	3	T58322	SC1.506	NDL 132, I-16	866177	3q22
LF111	3	BM005492	SC1.141	NDL 011, F-1	2185411	3p33*, multiple signals
LF128	3	BM005494	SC1.304	NDL 088, N-4	1395067	3p43
LF168	3	R47184	SC1.69	NDL 122, G-17	2946165	3q12
LF227	3	T58323	SC1.305	NDL 128, P-22	1379243	3p34
LF231	1	BM005478	SC1.1,1.62,1.88	NDL 027, C-12	4706946	3p23
LF232	3	BM005489	SC1.17	NDL 072, H-24	4314454	3p22
LF253	3	T58331	SC1.146,1.140	NDL 030, K-18	2147469	3p31
LF261	3	BM378052	SC1.901	NDL 047, B-12	389223	3q23
LF296	3	BM005501	SC1.179,1.798,1.191	NDL 014, J-19	1915443	3p14
LF316	3	BM005516	SC1.386	NDL 037, K-17	1156810	3p42*, multiple signals
LF323	3	BM005507	SC1.86	NDL 019, M-6	2716630	3q34
LF347	3	T58329	SC1.301	NDL 041, B-18	1424203	3p44
LF377	3	BM005496	SC1.13	NDL 089, F-18	4376253	3q44
LF396	3	BM005498	SC1.159	NDL 043, O-5	2117153	3p34
LF417	3	BM005499	SC1.150,1.483	NDL 087, E-8	2221698	3p33
LF92	3	BM005493	SC1.209	NDL 005, A-24	1779872	3p32*, multiple signals
LF96	3	BM005491	SC1.197	NDL 017, O-2	1881039	3p34
*MalI*	3	M30442	SC1.7,1.403	NDL 022, N-5	4891900	3p31
*PABP*	3	AY038043	SC1.470,1.253	NDL 008, N-7	908159	3q44
*para*	3	AF468968	SC1.186,1.312	NDL 056, P-6	1957664	3q25
*RpL31*	3	AF324863	SC1.217	NDL 008, B-14	1823077	3q34

Major signals are indicated by asterisks, NA – not applicable, LG – Linkage Group.

**Figure 3:**
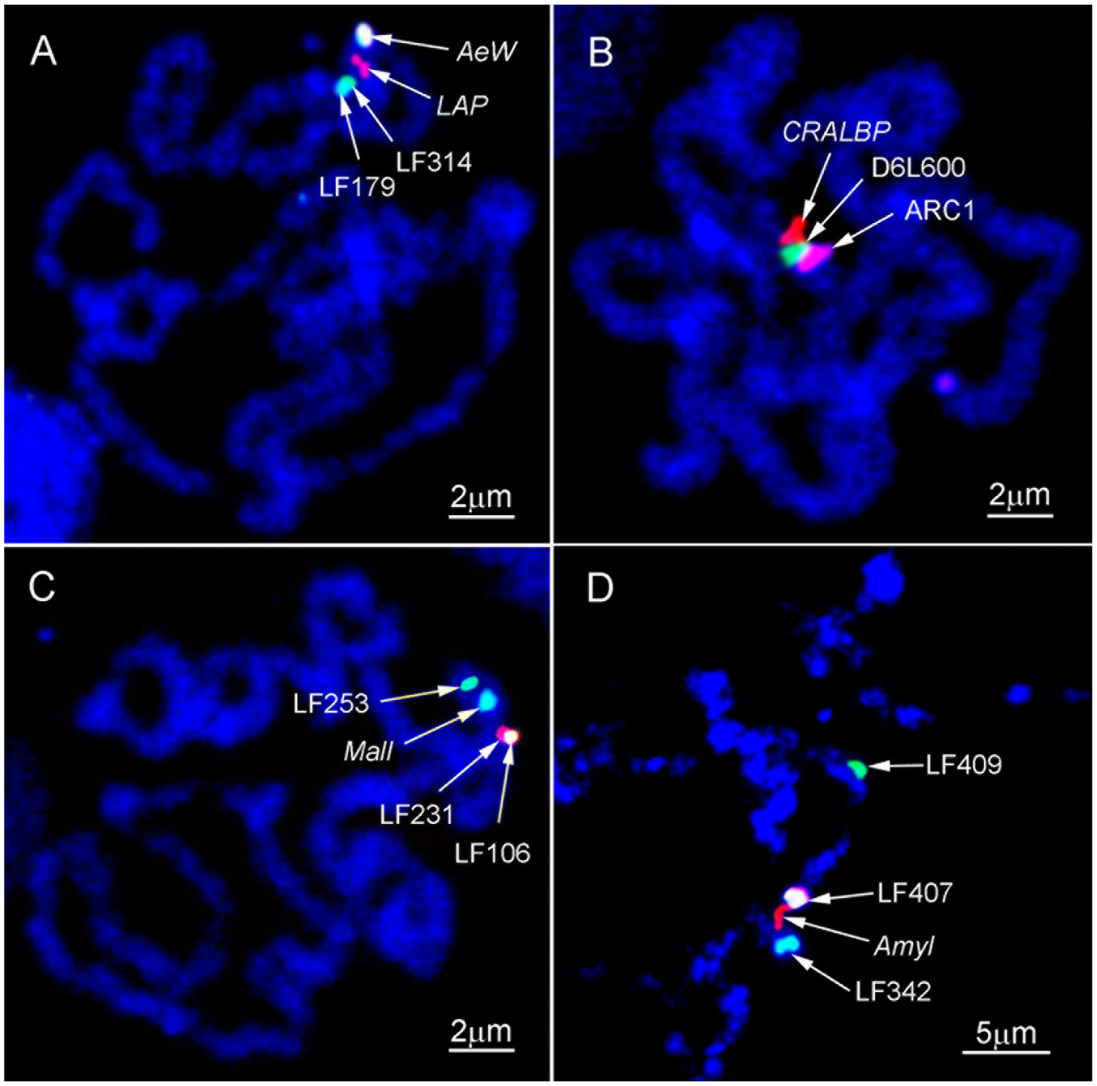
Multicolor FISH on mitotic and polytene chromosomes of *Aedes aegypti*. DNA probes labeled with fluorescein, Cy3, Cy5, and combinations of these dyes were hybridized to the prophase (A, B, C) and polytene chromosomes (D) stained by DAPI. The maximum resolutions of ∼0.5 Mb between markers LF179 and LF314 (A) and 300 kb between markers LF407 and *Amyl* (C) are obtained on prophase and polytene chromosomes, respectively.

**Figure 4:**
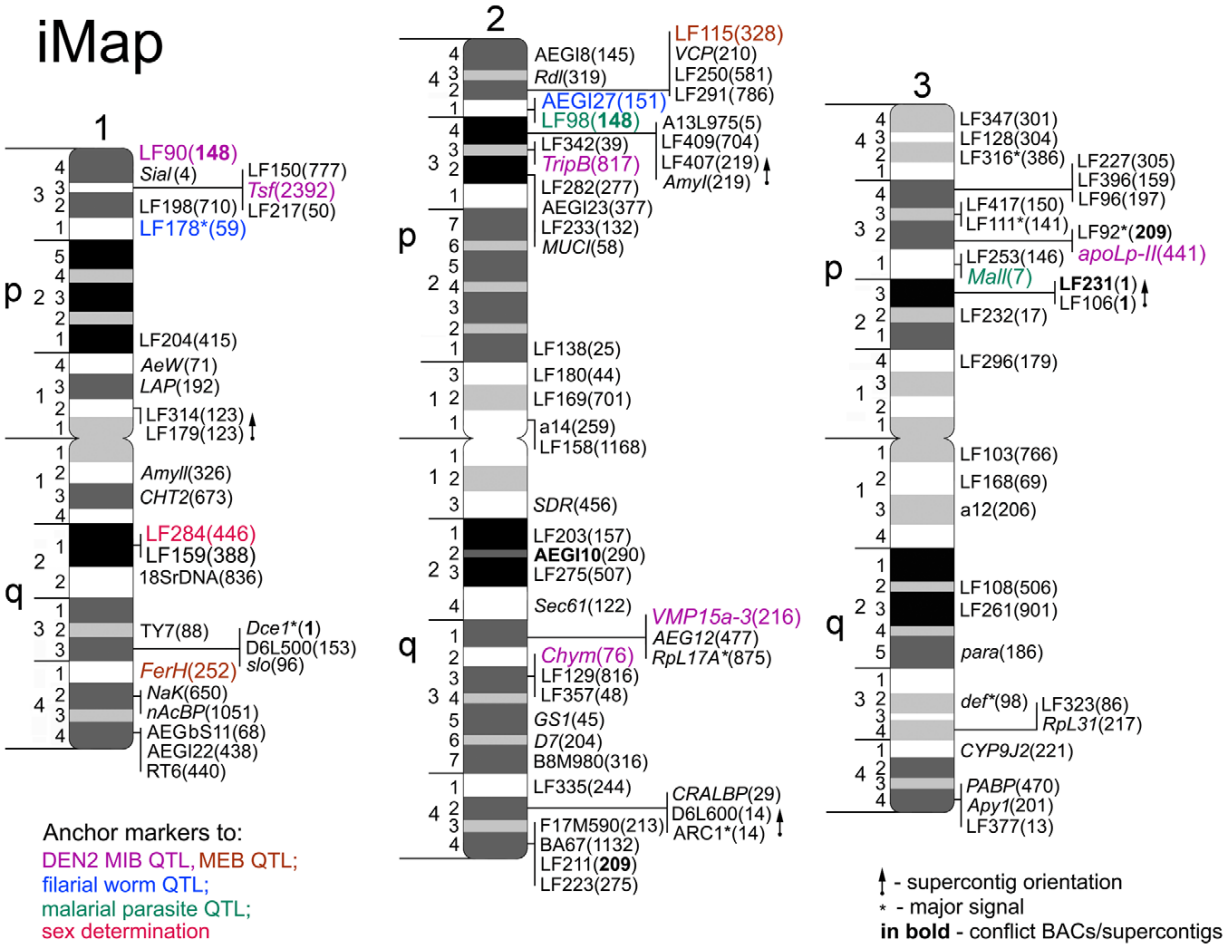
Integrated linkage, chromosome, and genome map–iMap of *Ae. aegypti*. Chromosome divisions and subdivisions are indicated on the left sides of the idiograms. The positions of genetic markers are shown on the right sides of the idiograms. The numbers of supercontigs in brackets are indicated by last 2–4 digits of supercontig ID. Anchor markers for dengue virus 2 (DEN2) midgut infection barrier (MIB) QTL are in purple; for DEN2 midgut escape barrier (MEB) QTL – in brown; filarial worm QTL - in blue; malaria parasite QTL – in green; for sex determination locus - in red. LF 98 is QTL anchor marker for both filarial worm and malaria parasite pathogens. The orientations of 4 supercontigs are demonstrated by arrows. Major locations of the BAC clones with multiple signals on the chromosomes are indicated by asterisks. Two BAC clones and 3 supercontigs in conflict with previous genetic mapping/genome assembly are in bold.

For ordering BAC clones within one band, FISH was performed on prophase chromosomes from imaginal discs or, for higher resolution, on polytene chromosomes from salivary glands of 4^th^ instar larvae of *Ae. aegypti*. Probes were labeled with three different dyes: fluorescein (green), Cy3 (red), and Cy5 (infrared), or with a combination of these dyes (Figure 3). Chromosomes after FISH were stained with DAPI (ultraviolet). This approach allowed mapping of up to 6 BAC clones simultaneously. Our FISH results showed that two probes have to be separated by a distance of ∼0.5 Mb on prophase chromosomes in order to be distinguished from each other (Figure 3A). The resolution of mapping using polytene chromosomes was even higher, ∼300 kb (Figure 3D). As a result of this additional mapping, all 100 BAC clones were placed in correct order on the chromosomes (Figure 4).

The physical map constructed in this study is the most populated physical map developed for *Ae. aegypti* thus far. Our current mapping effort placed 100 BAC clones and an 18S rDNA probe to their particular regions on the chromosomes. The “two-step” mapping approach significantly improved the resolution of the mapping. Using long prophase chromosomes and low-polytenized chromosomes from salivary glands, in addition to early metaphase chromosomes, provided the resolution similar to that obtained on polytene chromosomes from ovaries of *An. gambiae*, which is equal to ∼100 kb [44]. The current study developed a simple and robust technique for high-resolution physical mapping that can be further applied for more detailed physical mapping of the *Ae. aegypti* genome and other mosquito genomes. Similar to studies conducted on *Drosophila* [45]–[48], the physical mapping approach based on the banding patterns of mitotic chromosomes can also be used for the additional mapping of *An. gambiae* heterochromatin, which is under-replicated in normal polytene chromosomes.

### Integrating chromosome map with linkage and genome map of *Ae. aegypti*

**Figure 5:**
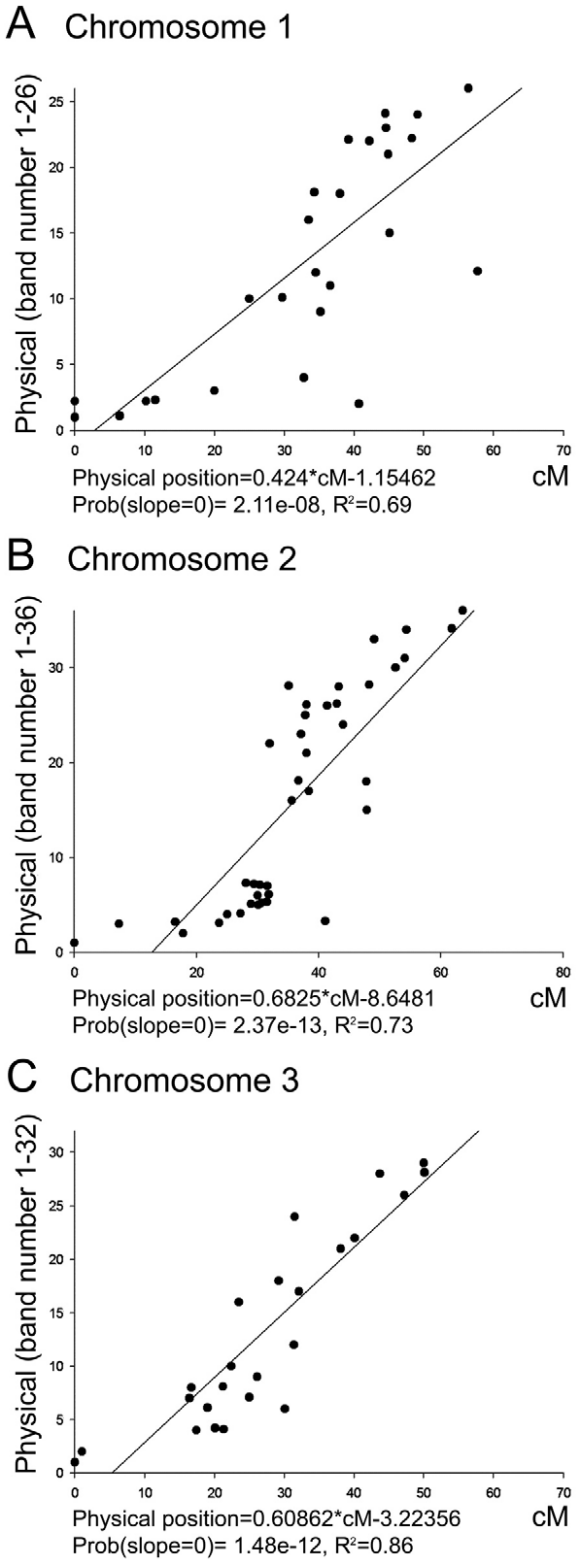
Linear regression analyses of the physical position of a gene as a function of its cM position. For chromosomes 1 (A), 2 (B), and 3 (C), the linear regression model is provided along with the probability that the slope is zero and the proportion of the total variance accounted for by the linear model (R^2^).

Mapping of the BAC clones that carry particular genetic markers allowed us to clarify the order of the genetic markers (Figure 4). Genetic markers physically mapped in this study span the entire chromosome complement. The longest chromosome 2 was the most densely populated with 45 genetic markers. The highest number of markers was found in areas close to the telomeres, especially on the p arm of chromosome 2. In contrast to the previous study [30], some markers were located around the centromeres. Surprisingly, two areas in the middle of the short arms on chromosome 1 and 2 (regions 1p2 and 2p2) had extremely low density of markers. A linear regression analysis demonstrated a good overall correlation between positions of the markers on the physical and linkage maps: R^2^ equaled to 0.69, 0.73, and 0.86 for chromosomes 1, 2, and, 3 respectively (Figure 5). On average, 1 cM on the linkage map corresponds to the half of a cytogenetic band or to 6.88 Mbs on the physical genome map. However, we found large discrepancies between the two maps with respect to the distances among markers located in areas near the centromeres and telomeres. These discrepancies probably caused by the high rate of recombination near telomeres and the low rate of recombination near the centromeres.

**Figure 6:**
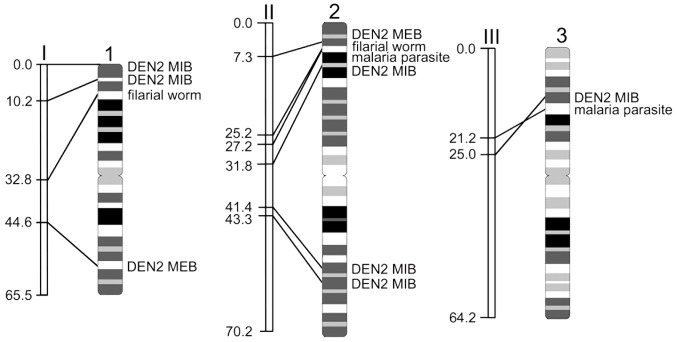
Localization of QTLs to various pathogens on linkage and chromosome maps of *Aedes aegypti*. The linkage positions of QTLs in cM on linkage groups I, II, and III are indicated on the left side. QTLs to dengue virus 2 (DEN2), midgut infection barrier (MIB), DEN2 midgut escape barrier (MEB), malaria parasite, and filarial worm are shown on the right sides of chromosomes 1, 2, and 3.

Based on previous studies, we were able to physycally map the positions of the QTLs related to the ability of *Ae. aegypti* to transmit different pathogens. The QTLs associated with the transmission of dengue virus 2 (DEN2) [19], [20]; filarioid nematode *Brugia malayi* [17] and the avian malaria parasite *Plasmodium gallinaceum* [15], [18] are indicated by different colors on Figure 4. As a result of physical mapping, 12 QTLs on the linkage map “collapsed” into five clusters of QTLs on the chromosome map in regions: 1p31–34; 2p33–42; 2q31–32 and 3p31–32 (Figure 6). Interestingly, four QTLs on 2p arm related to the transmission of various pathogens, such as filarioid nematode, avian malaria parasite, and dengue virus 2, were placed by different studies within a large region on the linkage map (31.8 cM or about 50% of chromosome 2). However, they were physically mapped within four chromosomal bands, which encompass only ∼11% of chromosome 2. These results suggest that the susceptibility of *Ae. aegypti* to diverse pathogens is controlled by fewer genomic loci than it was previously considered.

In addition to QTLs, the location of marker LF284, an anchoring marker for the sex determination locus [49], was also determined on chromosome 1 (Figure 4). This marker was localized in the intensively stained band in region 1q21. This region is located next to the ribosomal locus in negative band 1q22 that usually forms secondary constriction and can be easily identified on the chromosomes. Two BAC clones with markers AEGI10 and LF231 were found in conflict with previous mapping positions on different chromosomes. This result is not unexpected, as the genetic linkage map is a composite based on results of multiple independent genetic crosses [44].

Finally, the availability of the *Ae. aegypti* genome allowed us to map 100 genomic supercontigs to the chromosomes (Figure 4). Four supercontigs—1.123, 1.219, 1.14, and 1.1, which contained two or more genetic markers,—were oriented on the chromosomes. The orientation of these supercontigs is indicated by arrows on Figure 4. Physical mapping also helped us to identify potentially misassembled supercontigs if two or more BAC clones located in the same genomic supercontigs were mapped to different chromosomes. Our data suggests that three genomic supercontigs 1.148, 1.1, and 1.209 were misassembled in the previous study [31]. Potentially misassembled supercontigs are indicated in bold on Figure 4. In total, our mapping effort placed 183 Mb of genomic supercontigs, which is equal to 13.3% of the genome, to the chromosomes. The chromosome-based genome map for *Ae. aegypti* developed in this study is the second after the *An. gambiae* genome map developed for mosquitoes [32], [44], [50].

### Conclusion

The genomes of the three most dangerous for the human health species of mosquitoes—*Aedes aegypti*, *Anopheles gambiae*, and *Culex quinquefasciatus—*were sequenced in the last decade. The genome of *Ae. aegypti* is the largest among the three species and consists of 1,376 Mb. Our physical mapping effort incorporated 94 cytogenetic bands, 100 molecular genetic markers, and 183 Mb of the genome into one iMap of *Ae. aegypti*. The locations of anchor markers for QTLs related to dengue virus, filarial nematode, and malaria parasite transmission were determined on the chromosomes, as well as for the sex determination locus. Our discovery of the localization of multiple “unrelated” QTLs in a few major chromosome clusters suggests a possibility that the transmission of different pathogens is controlled by the same genomic loci. The study also demonstrated that physical mapping can orient genomic supercontigs and identify potential mistakes in genome assembly. Thus, the iMap developed here will facilitate the identification of genomic determinants of traits responsible for susceptibility or refractoriness of the mosquito to diverse pathogens and will also guide future efforts to improve the assembly of *Ae. aegypti* genome.
